# Robotic Versus Laparoscopic Versus Open Surgery for Non-Metastatic Pancreatic Neuroendocrine Tumors (pNETs): A Systematic Review and Network Meta-Analysis

**DOI:** 10.3390/jcm13216303

**Published:** 2024-10-22

**Authors:** Stelios-Elion Bousi, Marinos Zachiotis, Michail Papapanou, Maximos Frountzas, Dimitrios Symeonidis, Dimitrios Raptis, Basilios Papaziogas, Konstantinos Toutouzas, Evangelos Felekouras, Dimitrios Schizas

**Affiliations:** 1 First Department of Surgery, National and Kapodistrian University of Athens, Laikon General Hospital, 11527 Athens, Greece; stelbousi@gmail.com (S.-E.B.); mixalhspap13@gmail.com (M.P.); evangelosf@hotmail.com (E.F.); 2 First Propaedeutic Department of Surgery, National and Kapodistrian University of Athens, Hippocration General Hospital, 11527 Athens, Greece; froumax@hotmail.com (M.F.); tousur@hotmail.com (K.T.); 3 Department of Surgery, University Hospital of Larissa, 41110 Larissa, Greece; simeonid@hotmail.com; 4 Second Department of Surgery, Aristotle University of Thessaloniki, G. Gennimatas Hospital, 54635 Thessaloniki, Greece

**Keywords:** pancreatic surgery, neuroendocrine tumors, robotic surgery, laparoscopic surgery, minimally invasive surgery

## Abstract

**Background**: This systematic review, using pairwise and network meta-analyses, aimed to compare the intraoperative, short-term, and long-term postoperative outcomes of minimally invasive surgery (MIS) and open surgery (OS) for the management of pancreatic neuroendocrine tumors (pNETs). **Methods**: Studies reporting on the effects of robotic, laparoscopic, and open surgery on pNETs published before November 2023 on PubMed, Scopus, and CENTRAL were analyzed. **Results**: Thirty-two studies with 5379 patients were included in this review, encompassing 2251 patients undergoing MIS (1334 laparoscopic, 508 robotic, and 409 unspecified MIS) and 3128 patients undergoing OS for pNETs management. Pairwise meta-analysis revealed that the MIS group had a significantly shorter length of hospital stay ((a low certainty of evidence), MD of −4.87 (−6.19 to −3.56)); less intraoperative blood loss ((a low certainty of evidence), MD of −108.47 (−177.47 to −39.47)); and decreased tumor recurrence ((a high certainty of evidence), RR of 0.46, 95% CI (0.33 to 0.63)). Subgroup analysis indicated a higher R0 resection rate and prolonged operative time for laparoscopic surgery than for OS. The network meta-analysis ranked the robotic approach as superior in terms of the length of hospital stay, followed by the laparoscopic and OS arms. Furthermore, it favored both MIS approaches over OS in terms of the R0 resection rate. No significant differences were found in severe postoperative complications, postoperative fistula formation, mortality, readmission, reoperation, or conversion rates. **Conclusions**: This review supports the safety of MIS for the treatment of pNETs. However, the varying certainty of evidence emphasizes the need for higher-quality studies.

## 1. Introduction

The incidence of pancreatic tumors arising from islet cells, known as pancreatic neuroendocrine tumors (pNETs), has notably increased over the last few decades. This rise can be attributed primarily to significant advancements in both cross-sectional imaging and pathological evaluation tests [1]. Moreover, the updated TNM staging and WHO grading classifications contribute to achieving earlier and more accurate diagnoses [2,3,4]. However, pNETs still represent a mere 2% of total pancreatic neoplasms [5,6]. Surgical intervention remains the mainstay of the management for resectable sporadic and familial pNETs [7,8]. In this setting, minimally invasive pancreatic surgery has recently emerged to provide a feasible approach with potentially acceptable oncological results and lower morbidity rates [9,10].

Significant progress has been achieved since the first report of laparoscopic pancreatic resection for pNETs in 1996, shortly following the first laparoscopic pancreatic surgical procedure [11,12]. Another notable milestone in the development of minimally invasive surgery (MIS) for pNETs was the introduction of robotic surgery a few years later [13]. Surgical approaches ranging from minimally invasive resections to open pancreatectomies have since been employed for pNET treatment, and a considerable number of studies comparing these approaches have been documented in the literature [14,15,16,17,18,19,20,21,22,23,24,25,26,27,28,29,30,31,32,33,34,35,36,37,38,39,40,41,42,43,44,45].

Although previous meta-analyses have contributed greatly to the field, they have encountered limitations related to both the inherent characteristics of primary research publications within the field and systematic review methodology [46,47,48,49]. Among these constraints are deficiencies in the applied search strategy, a lack of clear and standardized data extraction criteria, and the absence of a comprehensive evaluation encompassing both the methodological quality of the included studies and the certainty of evidence associated with the outcomes of the meta-analysis. Acknowledging these limitations, there is a compelling need for a systematic review to address these methodological gaps. Furthermore, none of the previous systematic reviews compared robotic surgery, laparoscopic surgery, and open surgery (OS) arms.

The primary aim of this systematic review is to compare robotic, laparoscopic, and open surgery for pNETs across a wide range of intraoperative, immediate short-, and long-term postoperative outcomes. These outcomes were evaluated through both pairwise and network meta-analyses, taking advantage of both direct and indirect evidence from the existing literature.

## 2. Methods

### 2.1. Study Design and Protocol Registration

Our systematic review was conducted according to the PRISMA 2020 Statement Guidelines, ensuring a thorough and transparent approach to the identification, selection, data extraction, and synthesis of all pertinent studies. A predefined protocol was registered in the PROSPERO registry in January 2023 (registration number CRD42023390834) [50].

### 2.2. Eligibility Criteria

We included all published studies comparing MIS and OS for the treatment of pNETs. Retrospective and prospective studies published in English, German, or French were eligible, and no follow-up time limit was applied. The included studies were required to have a cumulative total of at least ten participants across all study arms. Studies were required to include only adult patients undergoing any type of pancreatic resection, such as enucleation, distal pancreatectomy (DP), pancreatoduodenectomy (PD), or central pancreatectomy, exclusively for the treatment of pNETs. If participants with other pathologies were also enrolled, this study was deemed eligible only if the outcomes of the patients with pNET were reported separately.

We excluded studies not satisfying the above criteria, as well as studies assessing non-surgical approaches, such as endoscopic management or chemotherapy. We decided to exclude all studies involving any patients with metastatic disease to maintain a more homogeneous population. We also excluded studies involving pediatric or pregnant patients. Case reports, conference abstracts, letters to the editor, and reviews were not considered to be eligible for inclusion.

### 2.3. Literature Search

We searched PubMed, Scopus, and CENTRAL databases from their inception until November 2023. No restrictions were imposed on the dates. We employed a meticulous search strategy to optimize the sensitivity of the search process. An extensive array of combinations of the terms “pancreatic neuroendocrine tumor”, “pancreatic resection”, and “minimally invasive surgery” and all their derivative terms were systematically generated and implemented to ensure a thorough exploration of the relevant literature.

Deduplication of the retrieved records was accomplished via a semiautomatic process using EndNote 20 (Clarivate Analytics, London, UK), and unique records were then imported into the Covidence platform [51,52]. Two independent blinded reviewers determined the compliance of each record with predefined eligibility criteria by sequentially reviewing the titles, abstracts, and full texts. Conflicts were resolved by a team consensus or by a third senior reviewer. Finally, references of the included studies, articles relevant to the included studies as returned by the “related articles” feature of PubMed search, and articles published by high-impact journals in the field were manually searched to identify any additional eligible studies (“snowball technique”).

### 2.4. Data Collection

Data extraction was conducted by two independent reviewers using a predefined Microsoft Excel spreadsheet (2016), which was agreed upon by all the authors. One reviewer performed the initial data extraction, and the other verified the accuracy and integrity of the extracted data.

The collected data encompassed a comprehensive range of information, including records and study characteristics, such as the first author, publication year, DOI, study center(s), study period, country of origin, study design, methods, number of total participants, per-group, and those lost to follow-up. Additionally, participant data, such as age, sex, BMI, and ASA score, were recorded. Pertinent details regarding pNET hormone production, pNET location, and characteristics of the intervention and comparators, including the pancreatectomy type, have also been documented. Outcome data were meticulously gathered, including the number of events and non-events in the intervention and comparator groups for each outcome, along with the adjusted effect measures in the multivariable regression models.

In studies that used methods to mitigate confounding bias, such as propensity score matching (PSM) and inverse probability of treatment weighting (IPTW), data extraction was conducted exclusively for information reported after the application of these techniques. Data on unmatched patients in these studies were not included in the subsequent analyses.

To facilitate subgroup analysis, in cases where studies reported outcomes separately for different MIS components, data on robotic and laparoscopic approaches were distinctly documented.

### 2.5. Outcomes

The primary outcomes encompassed the incidence of severe postoperative complications according to the Clavien–Dindo classification (grades 3–5) [53].

The secondary outcomes included length of hospital stay, R0 resection, recurrence rate, operative time, intraoperative blood loss, spleen preservation rate, postoperative infections, mortality, postoperative pancreatic fistula (POPF) formation (any grade), postoperative bleeding, readmission rate, reoperation rate, and conversion to OS (in the case of minimally invasive approaches).

### 2.6. Risk of Bias Assessment

Two blinded authors independently evaluated the risk of bias for each study. The Risk of Bias in Non-randomized Studies of Interventions (ROBINS-I) tool was exclusively used because all studies were non-randomized [54]. The studies were categorized into different levels of risk of bias, including low, moderate, serious, or critical, and were visualized using traffic light plots [55].

### 2.7. Statistical Analysis

#### 2.7.1. Summary of Measures, Assessment of Heterogeneity, and Pairwise Meta-Analysis

Categorical variables were presented as frequencies and percentages, whereas numerical variables were presented as mean (standard deviation) or median (range or interquartile range), depending on the normality of the data. Considering the clinical heterogeneity of the included population, we calculated the risk ratios and their respective 95% confidence intervals by fitting a random-effects model (DerSimonian-Laird) [56].

In our meta-analysis, variable N was used to represent the total number of participants included across all studies in each analysis. Whenever the N value was reported for each specific outcome, it was extracted and used in the subsequent analyses. In instances in which it was not explicitly reported, we assumed that the N value was equal to the total N of the entire arm of the study. Consequently, it was assumed that no patients were lost to follow-up during analysis.

For direct comparisons, a standard pairwise meta-analysis was performed. Inter-study heterogeneity was assessed using the *I*^2^ statistic. A value of *I*^2^ > 50% demonstrated substantial heterogeneity [57]. These were visualized by forest plots.

#### 2.7.2. Subgroup Analysis

For outcomes in which data regarding the various components of the MIS group were available, we performed subgroup analyses, directly comparing the specified MIS arms (robotic, laparoscopic, and unspecified MIS arm) with OS.

Considering recent data from large randomized controlled trials comparing MIS and open pancreatic surgery, notable variations in outcomes have emerged in the context of distal pancreatectomy (DP) versus pancreaticoduodenectomy (PD) [58,59]. Therefore, we planned a subgroup analysis for all procedure types (e.g., enucleation, DP, and PD) reported for the management of pNETs to address potential confounding bias.

#### 2.7.3. Sensitivity Analysis

To increase the robustness of our findings, we conducted sensitivity analyses that exclusively included studies with lower risk of bias. This involved excluding studies with either a serious risk of bias or both moderate and serious risks of bias to assess the impact of bias on our conclusions.

#### 2.7.4. Small Study Effects

Funnel plots were generated for all outcomes to assess the possibility of small effects. Additionally, for plots comprising a minimum of 10 studies, formal testing of asymmetry was conducted using Egger’s test, with significance indicated by *p* < 0.1 [60]. Upon detecting asymmetry in the funnel plot, contour-enhanced funnel plots were constructed to explore potential factors contributing to the observed asymmetry [56].

#### 2.7.5. Network Meta-Analysis

Connected networks of all three groups of robotic surgery, laparoscopic surgery, and OS were constructed for all outcomes, and their geometry was visualized [61]. The size of the nodes indicates the number of participants receiving each intervention, and the thickness of the lines between them represents the amount of direct evidence of the respective comparison. Global and local inconsistency tests were performed to statistically examine for transitivity [62]. The former was calculated based on the type of between-treatment comparison in all cases, and these values were subsequently utilized to test for global linearity through the Wald test, while the latter was based on White’s symmetrical alternative to the node-splitting method by Dias et al. [63,64,65]. Relative risks (after being back-transformed from the logarithmic scale) along with their 95% confidence intervals served as the measures of effect for all comparisons (*p* < 0.05 demonstrates statistical significance). The overall estimates of the effects and those pooled within each comparison were visualized using network forest and interval plots. Cumulative rankings for identifying superiority among the interventions were calculated and visualized through rankograms [66]. The treatment that has a higher cumulative probability of leading to a decrease in the risk for the investigated outcome is considered superior, except for the R0 resection rate. A network meta-analysis of the conversion rate to OS outcomes was not applicable.

All analyses were conducted in the Stata Statistical Software, version 13 (StataCorp LP, College Station, TX, USA) using the “metan” and “network” packages. A two-tailed *p*-value of less than 0.05 was considered statistically significant.

### 2.8. Certainty of Evidence

The evaluation of study quality and grading of recommendations were conducted using the “Grading of Recommendations Assessment, Development, and Evaluation” (GRADE) approach [67]. This established framework is employed to systematically evaluate the quality of evidence (QOE) in systematic reviews. GRADEpro software, a tool specifically designed for implementing the GRADE approach, was utilized to grade evidence and develop information for clinical practice [68].

## 3. Results

### 3.1. Search Strategy

**Figure 1:**
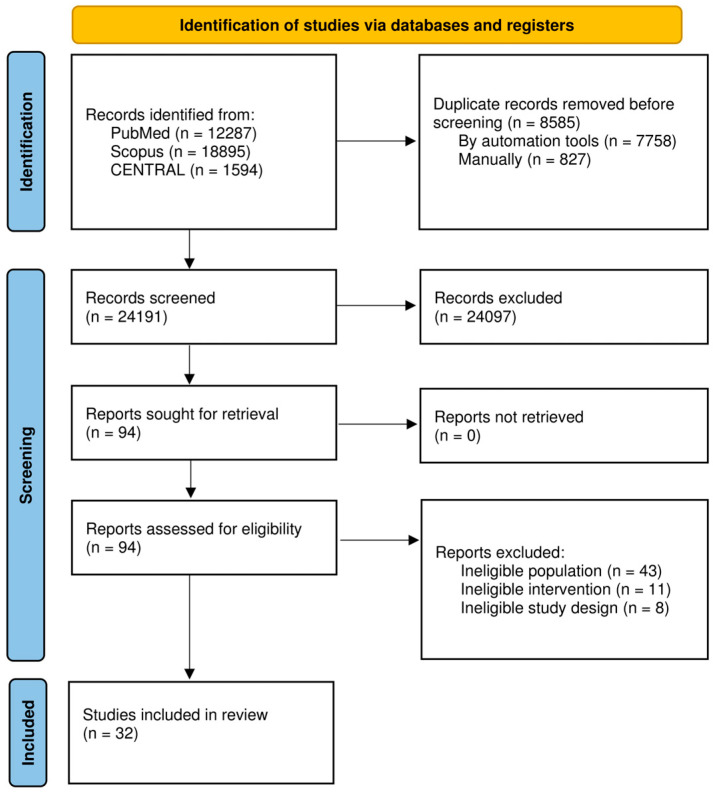
Preferred Reporting Items for Systematic Reviews and Meta-Analysis 2020 (PRISMA 2020) flow diagram of this study.

The literature search identified 32,776 potentially eligible publications. Following the deduplication process, 24,191 unique papers were retained. After screening titles and abstracts, 24,097 records were excluded as irrelevant. The remaining 94 articles were selected for full-text screening. Among them, 62 articles were subsequently excluded due to non-alignment with the eligibility criteria, resulting in the inclusion of 32 non-randomized controlled trials (NRCTs) that met the criteria. A schematic representation of the search algorithm is provided in the PRISMA flowchart (Figure 1).

### 3.2. Study Characteristics

In total, 5379 patients with pNETs were included in the study. Among them, 3128 (58.2%) underwent open surgical procedures, and 2251 (41.8%) underwent MIS. Among the latter, 1334 (24.8%) were planned for laparoscopic resection and 508 (9.4%) for robotic resection; the specific MIS component was not specified in 409 patients (7.6%). A total of 146 MIS procedures were identified as having been converted to open procedures. Two studies used the IPTW adjustment method, while four studies used the PSM method to control for the variables used [19,25,28,29,32,45]. The characteristics of the included studies are summarized in Table 1.

### 3.3. Risk of Bias Assessment

The included studies exhibited varying levels of risk of bias, with 5 (15.6%) classified as low, 17 (53.1%) as moderate, and 10 (31.3%) as a serious risk of bias (Supplementary Table S1).

### 3.4. Pairwise Meta-Analysis

**Figure 2:**
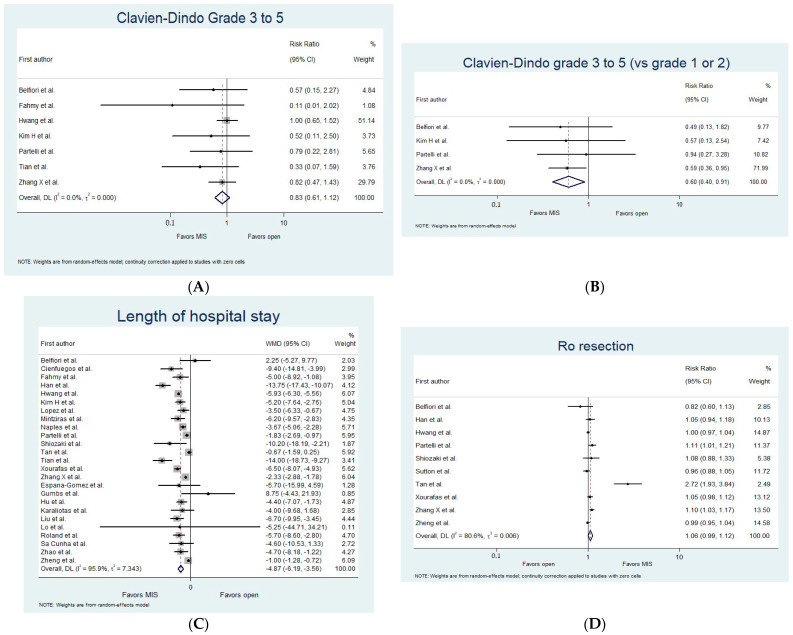

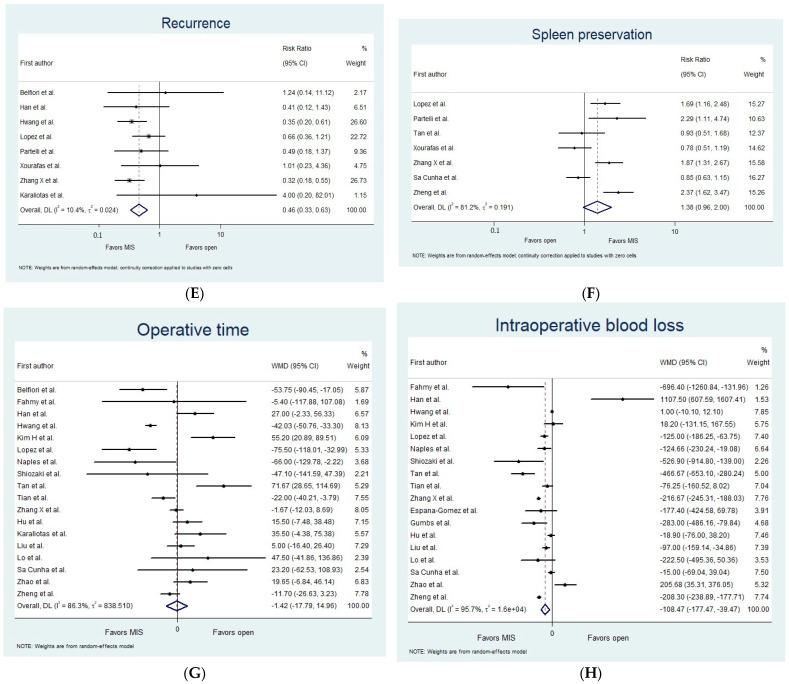

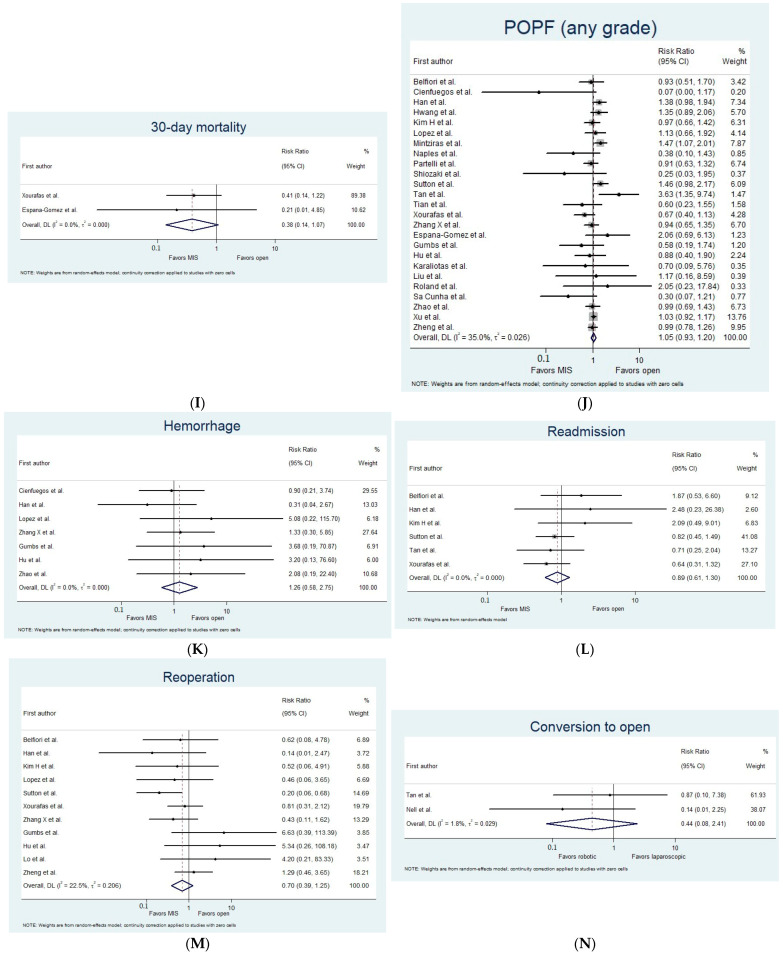
Forest plots of pairwise meta-analysis. (**A**) Severe postoperative complications according to Clavien–Dindo classification—grades 3 to 5 versus 0 to 2, (**B**) Severe postoperative complications according to Clavien–Dindo classification—grades 3 to 5 versus 1 to 2, (**C**) Length of hospital stay, (**D**) R0 resection, (**E**) Tumor recurrence, (**F**) Spleen preservation, (**G**) Operative time, (**H**) Intraoperative blood loss, (**I**) 30-day mortality, (**J**) Postoperative fistula formation, (**K**) Postoperative hemorrhage, (**L**) Readmission, (**M**) Reoperation, and (**N**) Conversion—robotic versus laparoscopic.

Forest plots of pairwise meta-analysis are shown in Figure 2. The limited number of studies providing separate outcome data for distinct procedure types, such as pancreaticoduodenectomy, enucleation, and distal pancreatectomy, hampers the significance of subgroup analyses. The findings of this subgroup analysis are presented in Supplementary Figure S1. Comparisons of the specified arms of MIS (robotic, laparoscopic, and unspecified MIS arms) with OS in outcomes for which relevant data were available are shown in Supplementary Figure S2.

#### 3.4.1. Primary Outcomes

##### Severe Complications According to the Clavien–Dindo Classification

There was no significant difference in the risk of severe complications, defined as Clavien–Dindo grades 3 to 5 (versus grades 0 to 2), between the MIS and the OS groups (RR 0.83, 95% CI (0.61 to 1.12), *I*^2^ = 0%, N = 1950, 7 studies, MIS vs. OS; Figure 2A) [15,17,19,20,25,29,32]. However, among the subset of patients who experienced any complications reported according to Clavien–Dindo classification (using the definition rate of a Clavien–Dindo grade of 1 or higher), the risk of those complications being severe (Clavien–Dindo grades 3 to 5 vs. grades 1 to 2) was significantly lower in patients who underwent MIS (RR 0.60, 95% CI (0.40 to 0.91), *I*^2^ = 0%, N = 334, 4 studies, MIS vs. OS; Figure 2B) [15,20,25,32].

#### 3.4.2. Secondary Outcomes

##### Length of Hospital Stay

We found that the length of hospital stay was significantly shorter in the MIS group (MD −4.87 (−6.19 to −3.56), *I*^2^ = 95.9%, N = 3992, 25 studies, MIS vs. OS; Figure 2C) [15,16,17,18,19,20,22,23,24,25,26,28,29,30,32,33,34,35,36,37,38,39,40,42,45]. Furthermore, our subgroup analysis revealed a shorter length of hospital stay (*p* interaction < 0.001) for both the laparoscopic and robotic subgroups compared to the open subgroup (MD −5.62 (−7.23 to −4.01), *I*^2^ = 79.4%, N = 1483, 17 studies, laparoscopic vs. OS; Supplementary Figure S2C) [16,18,20,23,24,25,26,30,33,34,35,36,37,38,39,40,42] and (MD −14.00 (−18.73 to −9.27), *I*^2^ = 0%, N = 120, 1 study, robotic vs. OS; Supplementary Figure S2C) [29].

##### R0 Resection

No significant difference was observed in R0 resection between the MIS and OS arms (RR 1.06, 95% CI (0.99 to 1.12), *I*^2^ = 80.6%, N = 3360, 10 studies, MIS vs. OS; Figure 2D) [15,18,19,25,26,27,28,30,32,45]. However, subgroup analysis revealed a significantly higher probability of R0 resection for patients who underwent laparoscopic surgery (RR 1.07, 95% CI (1.02 to 1.12), *I*^2^ = 0%, N = 415, 4 studies, laparoscopic vs. OS; Supplementary Figure S2D) [18,25,26,30].

##### Tumor Recurrence

A significantly lower risk of tumor recurrence was observed in the MIS group (RR 0.46, 95% CI (0.33 to 0.63), *I*^2^ = 10.4%, N = 1646, 8 studies, MIS vs. OS; Figure 2E) [15,18,19,22,25,30,32,36].

##### Spleen Preservation

There was no significant difference in the spleen preservation rate (RR 1.38, 95% CI (0.96 to 2.00), *I*^2^ = 81.2%, N = 2018, 7 studies, MIS vs. OS; Figure 2F) [22,25,28,30,32,40,45]. However, our sensitivity analysis revealed a higher spleen preservation rate in the MIS arm, both when excluding the studies considered to be of serious risk of bias (RR 1.78, 95% CI (1.35 to 2.35), *I*^2^ = 45.6%, N = 1826, 5 studies, MIS vs. OS) [22,25,28,32,45] and when considering only a low risk of bias studies after excluding those with a moderate and serious risk of bias (RR 1.69, 95% CI (1.07 to 2.68), *I*^2^ = 70.7%, N = 1669, 3 studies, MIS vs. OS) [28,32,45].

##### Operative Time

No significant difference in the operative time was found between the MIS and OS groups (MD −1.42 (−17.79 to 14.96), *I*^2^ = 86.3%, N = 3520, 18 studies; Figure 2G) [15,17,18,19,20,22,24,26,28,29,32,35,36,37,38,40,42,45]. However, a significant difference between subgroups was observed (*p* interaction = 0.002), with the laparoscopic subgroup exhibiting an increased operative time compared to the open subgroup (MD 18.00 (2.01 to 33.98), *I*^2^ = 43.8%, N = 720, 10 studies, laparoscopic vs. OS; Supplementary Figure S2G) [18,20,24,26,35,36,37,38,40,42] and the robotic subgroup exhibiting a decreased operative time (MD −22.00 (−40.21 to −3.79), *I*^2^ = 0%, N = 120, 1 study, robotic vs. OS; Supplementary Figure S2G) [29].

##### Intraoperative Blood Loss

Patients who underwent minimally invasive pancreatic resection also experienced significantly less intraoperative blood loss (MD −108.47 (−177.47 to −39.47), *I*^2^ = 95.7%, N = 3505, 18 studies, MIS vs. OS; Figure 2H) [17,18,19,20,22,24,26,28,29,32,33,34,35,37,38,40,42,45].

##### Postoperative Mortality

No significant differences were observed in the postoperative mortality. A total of 2 studies reported results for laparoscopic vs. OS 30-day postoperative mortality (RR 0.39, 95% CI (0.14 to 1.07), *I*^2^ = 0%, N = 205, 2 studies; Figure 2I) [30,33], and 1 study reported results for robotic vs. OS 90-day postoperative mortality (RR 0.33, 95% CI (0.01 to 8.02), N = 120, 1 study) [29].

##### Postoperative Pancreatic Fistula (POPF) Formation and Postoperative Hemorrhage

There were no significant differences observed in the risk of POPF formation (RR 1.05, 95% CI (0.93 to 1.20), *I*^2^ = 35.0%, N = 4340, 25 studies, MIS vs. OS; Figure 2J) [15,16,18,19,20,22,23,24,25,26,27,28,29,30,32,33,34,35,36,37,39,40,42,44,45] and postoperative hemorrhage (RR 1.26, 95% CI (0.58 to 2.75), *I*^2^ = 0%, N = 845, 7 studies, MIS vs. OS; Figure 2K) [16,18,22,32,34,35,42].

##### Readmission and Reoperation Rate

We did not observe any significant differences in the risk of readmission (RR 0.89, 95% CI (0.61 to 1.30), *I*^2^ = 0%, N = 714, 6 studies, MIS vs. OS; Figure 2L) [15,18,20,27,28,30] and reoperation rates (RR 0.70, 95% CI (0.39 to 1.25), *I*^2^ = 22.5%, N = 2113, 11 studies, MIS vs. OS; Figure 2M) [15,18,20,22,27,30,32,34,35,38,45].

##### Conversion Rate (Among MIS Approaches)

Similarly, there was no significant difference in the risk of conversion to OS between the laparoscopic and robotic approaches (RR 0.44, 95% CI (0.08 to 2.41), *I*^2^ = 1.8%, N = 57, 2 studies, robotic vs. laparoscopic; Figure 2N) [28,43].

### 3.5. Small Study Effects

Significant small-study effects were not observed, except for the Clavien–Dindo classification of postoperative complications, recurrence rate, readmission rate, and postoperative hemorrhage. Visual assessment was employed to evaluate the small study effect in these specific outcomes because of the limited number of available studies (less than 10), rendering Egger’s test inapplicable. The small study effects on the above outcomes may be attributed to publication bias, as indicated by the contour-enhanced funnel plots. For the remaining outcomes that met the criteria for the applicability of Egger’s test, no indications of asymmetry were observed, suggesting the absence of publication bias. Generated funnel and contour-enhanced funnel plots are provided in Supplementary Figures S3 and S4, respectively.

### 3.6. Certainty of Evidence

A summary of the findings regarding the certainty of evidence for the primary and secondary outcomes is shown in Table 2.

#### 3.6.1. Primary Outcomes

The occurrence of severe postoperative complications according to the Clavien–Dindo classification (graded 3–5 versus 0–2) was assessed as having a very low certainty of evidence.

#### 3.6.2. Secondary Outcomes

Recurrence was assessed to have a high certainty of evidence. The length of hospital stay and intraoperative blood loss outcomes were classified as having a low certainty of evidence, whereas the certainty of evidence regarding the operative time, R0 resection, and POPF formation outcomes was determined to be very low. The certainty of evidence regarding the length of hospital stay and intraoperative blood loss was further downgraded due to inconsistency (*I*^2^ = 95.9% and *I*^2^ = 95.7%, respectively), whereas the certainty of evidence regarding POPF formation was further downgraded due to imprecision (95% CI, 0.93 to 1.20). The certainty of evidence for R0 resection (95% CI, 0.99 to 1.12; *I*^2^ = 80.6%) and operative time (MD −17.79 to 14.96; *I*^2^ = 86.3%) were further downgraded due to both imprecision and inconsistency.

### 3.7. Network Meta-Analysis

**Figure 3:**
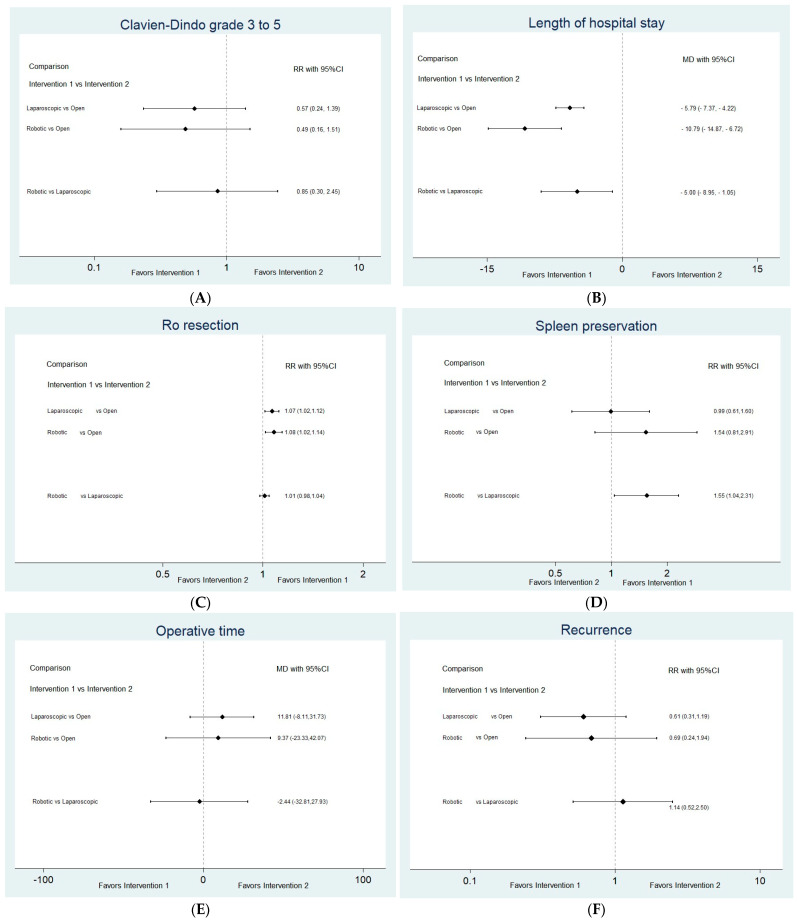

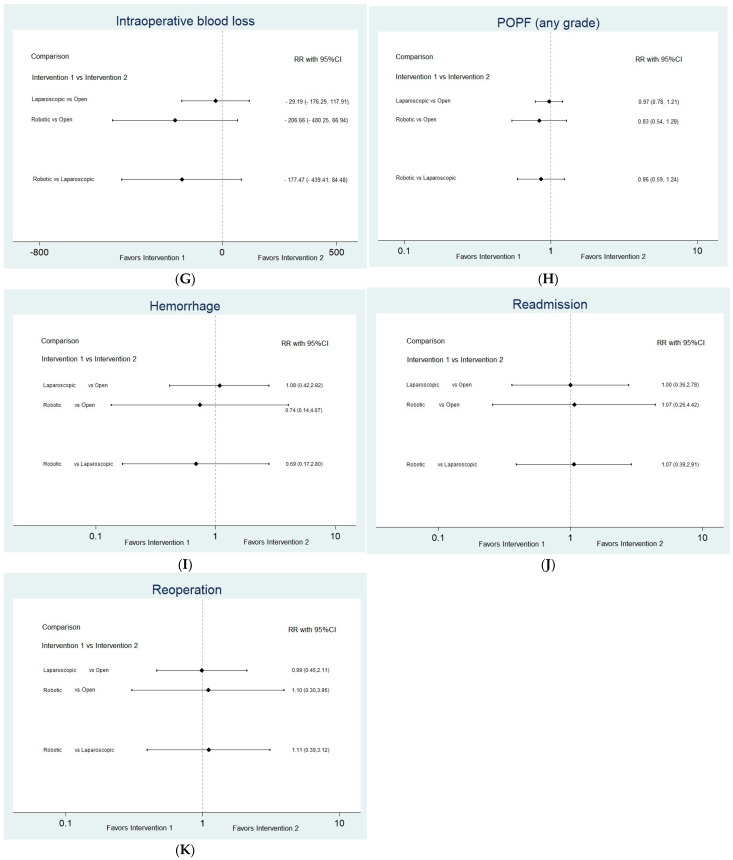
Interval plots of network meta-analysis. (**A**) Severe complications according to Clavien–Dindo classification—grades 3 to 5 versus 0–2, (**B**) Length of hospital stay, (**C**) R0 resection, (**D**) Spleen preservation, (**E**) Operative time, (**F**) Tumor recurrence, (**G**) Intraoperative blood loss, (**H**) Postoperative fistula formation, (**I**) Postoperative hemorrhage, (**J**) Readmission, and (**K**) Reoperation.

Interval plots from the network meta-analysis are shown in Figure 3. We accepted the consistency hypothesis for all outcomes except for the operative time and spleen preservation rates, for which the *p*-value of the global test for inconsistency was less than 0.05. The structures of the connected networks for all the investigated outcomes are shown in Supplementary Figure S5. Forest plots are shown in Supplementary Figure S6.

#### 3.7.1. Primary Outcomes

##### Severe Complications According to the Clavien–Dindo Classification

No significant difference was found in the risk of severe complications, defined as Clavien–Dindo grades 3 to 5 (versus grades 0–2) between any of the arms (laparoscopic vs. OS RR 0.57, 95% CI (0.24 to 1.39); robotic vs. OS RR 0.49, 95% CI (0.16 to 1.51); robotic vs. laparoscopic RR 0.85, 95% CI (0.30 to 2.45), N = 574, 4 studies; Figure 3A) [14,20,25,29].

#### 3.7.2. Secondary Outcomes

##### Length of Hospital Stay

A significant difference in the length of hospital stay was observed among the different intervention groups (laparoscopic vs. OS MD −5.79 (−7.37, −4.22); robotic vs. OS MD −10.79, 95% CI (−14.87 to −6.72); robotic vs. laparoscopic MD −5.00, 95% CI (−8.95 to −1.05), N = 1698, 20 studies; Figure 3B) [16,18,20,23,24,25,26,29,30,31,33,34,35,36,37,38,39,40,42,43].

##### R0 Resection

We also found a significantly higher R0 resection rate in both the robotic and laparoscopic groups than in the open group; however, no significant difference was detected between the two MIS approaches (laparoscopic vs. OS RR 1.07, 95% CI (1.02 to 1.12); robotic vs. OS RR 1.08, 95% CI (1.02 to 1.14); robotic vs. laparoscopic RR 1.01, 95% CI (0.98 to 1.04), N = 617, 6 studies; Figure 3C) [14,18,25,26,30,43].

##### Spleen Preservation

There was a significant difference in the spleen preservation rate between robotic and laparoscopic surgery, while no significant difference was observed when comparing MIS approaches with OS (laparoscopic vs. OS RR 0.99, 95% CI (0.61 to 1.60), robotic vs. OS RR 1.54, 95% CI (0.81 to 2.91), robotic vs. laparoscopic RR 1.55, 95% CI (1.04 to 2.31), N = 571, 5 studies; Figure 3D) [14,25,30,31,40]. In this outcome, the global test for incoherence indicated significant inconsistency (*p* < 0.05).

##### Other Outcomes

No significant differences were observed in the operative time (Figure 3E), tumor recurrence rate (Figure 3F), intraoperative blood loss (Figure 3G), POPF formation (Figure 3H), postoperative hemorrhage (Figure 3I), readmission rate (Figure 3J), and reoperation rate (Figure 3K).

#### 3.7.3. Ranking of Treatments

The relative ranking of the three surgical approaches, based on the estimation of cumulative ranking probabilities and visualized through rankograms, along with pertinent local inconsistency tests, are shown in Supplementary Figure S7. These findings should be interpreted in conjunction with the reported effect estimates.

## 4. Discussion

### 4.1. Summary of Findings

This review demonstrated that there was no discernible difference observed between MIS and OS in the occurrence of severe postoperative complications (classified as Clavien–Dindo grades 3 to 5 versus grades 0 to 2). With a certainty of evidence downgraded further due to imprecision, this outcome suggests that MIS may reduce severe complications compared to OS. However, this outcome was deemed to have a very low certainty of evidence.

Within the subset of patients who develop postoperative complications (Clavien–Dindo grades ≥ 1) during surgery for pNETs, those who undergo MIS have a significantly reduced risk of experiencing severe complications (classified as Clavien–Dindo grades 3 to 5 versus grades 1 to 2).

MIS also appears to be associated with a shorter hospital stay, with the robotic subgroup possibly exhibiting an even shorter hospital stay than that of the laparoscopic subgroup. Having a low certainty of the evidence, MIS may reduce the length of hospital stay. This finding from the pairwise meta-analysis aligns with the results of the network meta-analysis.

There was no significant difference in R0 resection rates between the MIS and OS groups. With a very low certainty of evidence, this outcome suggests that MIS may have little or no effect on R0 resection; however, the evidence is uncertain. Both pairwise subgroup analysis and network meta-analysis suggest that the laparoscopic approach may contribute to a higher R0 resection rate. Moreover, the network meta-analysis, based on indirect evidence, indicated that the robotic approach could also result in higher R0 rates than OS.

A significant reduction in the recurrence rate of pNETs was observed in MIS patients. The high certainty of the evidence for this outcome suggests that MIS decreases the recurrence rate.

Notably, a meta-analysis of studies with a lower risk of bias confirmed that the spleen preservation rate was significantly higher in the MIS group. The network meta-analysis favored the robotic over laparoscopic approach but did not show an advantage for MIS arms over the OS arm. However, the latter finding is marked by a high degree of inconsistency.

There was no significant difference between the MIS and OS arms in terms of the operative time. However, our subgroup analysis revealed a longer operative time for the laparoscopic approach and a shorter operative time for the robotic approach than for OS. With a very low certainty of evidence, this outcome suggests that MIS may have little or no effect on the operative time; however, the evidence is very uncertain.

There was a significant reduction in intraoperative blood loss for MIS compared to OS for pNETs. This outcome suggests that MIS may reduce intraoperative blood loss, but the certainty of evidence is low.

All other comparisons did not demonstrate a significant difference between the intervention groups.

### 4.2. Comparison with the Literature and Clinical Significance

A significant number of primary studies on this topic have been conducted since the publication of the latest relevant systematic reviews [17,19,21,24,25,26,27,44,45]. In this study, we included nearly twice as many original studies and nearly three times the number of patients compared to the most recent systematic review [49]. This demonstrates both the expansion of the reported research efforts and the more comprehensive scope of the literature search carried out in this study.

#### 4.2.1. Intraoperative Outcomes

In terms of intraoperative outcomes, we found that the MIS approach reduces blood loss, primarily because of the advantages of smaller incisions and precision in dissecting tissues and blood vessels that MIS procedures can provide [69]. In contrast to all four previous systematic reviews, our findings suggest that laparoscopic surgery seems to increase the operative time. This disparity can be partially attributed to the inclusion of studies that considered converted operations to laparoscopic procedures. In our analysis, we categorized these procedures as MIS using an intention-to-treat methodology. These findings align with those of a recent meta-analysis of large RCTs comparing minimally invasive and open pancreatic surgery [70]. Concerning spleen preservation, most recent guidelines suggest that both minimally invasive approaches are feasible options, with robotic surgery having similar or higher rates of spleen preservation, in alignment with our network meta-analysis findings [71,72]. This systematic review is the first to compare laparoscopic and robotic approaches for pNETs in terms of conversion rates. Among the previous systematic reviews, the latest was the only one that included robotic procedures for pNETs without considering them as a distinct arm [49]. This systematic review is the first to compare laparoscopic and robotic approaches for pNETs, revealing no significant difference between their conversion rates.

#### 4.2.2. Short-Term Postoperative and Histopathological Outcomes

Our findings regarding short-term postoperative complications suggest that the minimally invasive nature of a surgical operation is potentially beneficial in mitigating the severity of these complications. Specifically, the MIS group exhibited a significantly lower risk of complications, deemed as Clavien–Dindo grades 3 to 5 versus grades 1 to 2 compared to the OS group. While there were no significant differences between these approaches for most of our short-term postoperative findings, this superiority may be due to additional complications not captured in our secondary outcomes, which may have further favored MIS. These outcomes could further be linked to the reduced length of hospital stay, which is consistent with previous systematic reviews and corroborated by the most updated literature [46,47,48,49,72]. It is worth noting that in terms of hospital stay duration, our network meta-analysis ranked the robotic approach as superior, followed by laparoscopic surgery and OS arms. Although the literature suggests that parenchyma-sparing MIS procedures, commonly used for pNETs, may carry a risk of POPF formation, our meta-analysis did not reveal any significant differences between distinct surgical approaches [45,73]. Regarding other short-term postoperative outcomes, such as postoperative hemorrhage, readmission, and reoperation rates, our findings agree with those of all previous systematic reviews, suggesting no clear predominance of any surgical approach [46,47,48,49]. Only a limited number of original studies have reported the incidence of postoperative mortality in the context of surgery for pNETs. Based on these sparse data, no clear predominance of any surgical approach has been established. Our network meta-analysis underscores the superiority of both MIS arms over OS in achieving higher R0 resection rates, primarily owing to the meticulous nature of MIS procedures, which, in turn, contributes to a reduced risk of local recurrence.

#### 4.2.3. Long-Term Postoperative Outcomes

The lower recurrence rate observed in the MIS group could be attributed to the enhanced precision and effectiveness associated with MIS approaches. These findings align with the conclusions of the latest systematic review [49]. However, it is crucial to consider the impact of prognostic parameters, such as the WHO classification and TNM stage of pNETs, on oncological results [74]. In contrast to intraoperative or short-term postoperative outcomes, the evaluation of the oncological outcomes necessitates a separate analysis for pNETs compared with pancreatic adenocarcinomas. This differentiation is essential because of the distinctive characteristics of pNETs, as opposed to those of adenocarcinomas, including observed variations in the levels, routes, and frequencies of lymph nodal metastases between these tumor types [75].

### 4.3. Strengths and Limitations

To our knowledge, this is the largest systematic review and the first to compare all possible arms through a frequentist network meta-analysis, including both OS and MIS for pNETs, while also separately encompassing laparoscopic and robotic arms in cases where the MIS component was specified.

However, some studies lacked separate data on MIS approaches, such as laparoscopic or robotic surgery, resulting in smaller sample sizes. To overcome this limitation, we extracted all subgroup data when reported separately, which allowed us to obtain both direct and indirect evidence through our meta-analysis.

We compared the three surgical approaches across the widest range of outcomes possible. However, other outcomes, such as the risk of postoperative infection or the postoperative development of diabetes mellitus, were assessed in very few, if any, of the included studies. Additionally, more long-term outcomes, such as quality of life, were only scarcely reported in the analyzed studies.

To further investigate heterogeneity, we conducted subgroup analyses according to the procedure type. Due to limited data, it was impossible to conduct additional sensitivity analyses for other potential effect modifiers, such as tumor grade, tumor stage, tumor functionality, the implementation of adjuvant or neoadjuvant therapies, center resources, and surgeons’ experience. Additionally, only a few studies included patients with familial pNETs, making it infeasible to extract separate conclusions for this subgroup.

Despite our exhaustive literature search, we did not identify any RCTs, leading to a meta-analysis consisting solely of observational studies. Furthermore, a significant proportion of the included studies carried a serious risk of bias. To assess the robustness of our findings, we performed sensitivity analyses focusing on studies with a low risk of bias. However, only a few included studies met this criterion.

Our systematic review was the first to implement the GRADE approach. However, the certainty of evidence regarding outcomes varied from high to very low and was occasionally downgraded owing to the limitations of the included studies, inconsistency, and/or imprecision.

## 5. Conclusions

This systematic review and meta-analysis revealed a significantly shorter length of hospital stay (low certainty of evidence), less intraoperative blood loss (low certainty of evidence), and decreased recurrence rate (high certainty of evidence) in patients undergoing MIS than in those undergoing open procedures for pancreatic neuroendocrine tumors. Furthermore, the network meta-analysis suggested that both laparoscopic and robotic surgery can reduce the length of hospital stay and increase R0 resection rates compared with OS. These findings suggest that MIS is safe and feasible for the treatment of pancreatic neuroendocrine tumors in centers with sufficient experience. To conduct a more comprehensive evaluation and obtain more robust conclusions in the future, it is mandatory to have larger, higher-quality observational studies or, ideally, RCTs with separate data for open, laparoscopic, and robotic approaches.

## Figures and Tables

**Table 1:** Summary of characteristics of included studies. pNETs: pancreatic neuroendocrine tumors; N/A: not available. MEN 1: multiple endocrine neoplasia type 1; IPTW: Inverse Probability Treatment Weighting; PSM: propensity score matching. Studies are summarized in Table 1.

Study	Year	Study Design	Country	Patients (n)	Type(s) of Surgical Procedure	Compared Surgical Approaches	Surgical Approach (n)			
							OS	Laparoscopic	Robotic	MIS
Alfieri et al. [14]	2019	Multicenter Retrospective cohort	Italy	181	Distal pancreatectomy	Robotic vs. Laparoscopic Surgery	N/A	85	96	N/A
Belfiori et al. [15]	2018	Multicenter Retrospective cohort	Italy and Germany	71	Enucleation	MIS vs. OS	56	12	3	15
Cienfuegos et al. [16]	2016	Single center Retrospective cohort	Spain	79	Enucleation Central pancreatectomy Distal pancreatectomy Pancreaticoduodenectomy	Laparoscopic vs. OS	43	36	N/A	N/A
Fahmy et al. [17]	2021	Single-center Retrospective cohort	USA	87	Enucleation Distal pancreatectomy	MIS vs. OS	56	16	15	31
Han et al. [18]	2017	Single-center Retrospective cohort	South Korea	94	Distal pancreatectomy	Laparoscopic vs. OS	52	42	N/A	N/A
Hwang et al. [19]	2021	Multicenter Retrospective cohort IPTW	South Korea	859	Enucleation Central pancreatectomy Distal pancreatectomy Pancreaticoduodenectomy Total pancreatectomy	MIS vs. OS	478	356	25	381
Kim H et al. [20]	2019	Single-center Retrospective cohort	South Korea	149	Pancreaticoduodenectomy	Laparoscopic vs. OS	91	58	N/A	N/A
Kim J et al. [21]	2020	Single-center Retrospective cohort	South Korea	110	Enucleation Distal pancreatectomy Pancreaticoduodenectomy	MIS vs. OS	48	47	15	62
Lopez et al. [22]	2016	Single-center Retrospective cohort	Germany	33	Enucleation Distal pancreatectomy	MIS vs. OS	21	8	4	12
Mintziras et al. [23]	2019	Multicenter Retrospective cohort	Germany	287	Enucleation Distal pancreatectomy Pancreaticoduodenectomy Total pancreatectomy	Laparoscopic vs. OS	237	50	N/A	N/A
Naples et al. [24]	2022	Single-center Retrospective cohort	USA	34	Enucleation Distal pancreatectomy Pancreaticoduodenectomy	Laparoscopic vs. OS	23	11	N/A	N/A
Partelli et al. [25]	2021	Single-center Retrospective cohort IPTW	Italy	124	Distal pancreatectomy	MIS vs. OS	84	40	N/A	N/A
Shiozaki et al. [26]	2021	Single-center Retrospective cohort	Japan	26	Distal pancreatectomy	Laparoscopic vs. OS	13	13	N/A	N/A
Sutton et al. [27]	2022	Multicenter Retrospective cohort	USA	282	Enucleation Distal pancreatectomy Pancreaticoduodenectomy Total pancreatectomy	MIS vs. OS	139	NR	NR	143
Tan et al. [28]	2020	Single-center Retrospective cohort PSM	Singapore	134	Enucleation Distal pancreatectomy Pancreaticoduodenectomy Total pancreatectomy	MIS vs. OS	98	26	10	36
Tian et al. [29]	2016	Single-center Case-control study PSM	China	120	Enucleation	MIS vs. OS	60	N/A	60	N/A
Xourafas et al. [30]	2015	Single-center Retrospective cohort	USA	171	Distal pancreatectomy	Laparoscopic vs. OS	98	73	N/A	N/A
Zhang J et al. [31]	2017	Single-center Retrospective cohort	China	74	Distal pancreatectomy	Robotic vs. Laparoscopic Surgery	N/A	31	43	N/A
Zhang X et al. [32]	2019	Multicenter Retrospective cohort PSM	USA	576	Distal pancreatectomy	MIS vs. OS	362	NR	NR	214
Espana-Gomez et al. [33]	2009	Single-center Retrospective cohort	Mexico	34	Enucleation Central pancreatectomy Distal pancreatectomy Pancreaticoduodenectomy	Laparoscopic vs. OS	13	21	N/A	N/A
Gumbs et al. [34]	2008	Single-center Retrospective cohort	France	31	Enucleation Central pancreatectomy Distal pancreatectomy Pancreaticoduodenectomy	Laparoscopic vs. OS	13	18	N/A	N/A
Hu et al. [35]	2011	Single-center Retrospective cohort	China	89	Enucleation Distal pancreatectomy	Laparoscopic vs. OS	46	43	N/A	N/A
Karaliotas et al. [36]	2009	Single-center Retrospective cohort	Greece	12	Enucleation	Laparoscopic vs. OS	7	5	N/A	N/A
Liu et al. [37]	2007	Multicenter Retrospective cohort	China	48	Enucleation Central pancreatectomy Distal pancreatectomy Pancreaticoduodenectomy	Laparoscopic vs. OS	41	7	N/A	N/A
Lo et al. [38]	2004	Single-center Retrospective cohort	China	10	Enucleation Distal pancreatectomy	Laparoscopic vs. OS	6	4	N/A	N/A
Roland et al. [39]	2008	Multicenter Retrospective cohort	USA and China	37	Enucleation Distal pancreatectomy	Laparoscopic vs. OS	15	22	N/A	N/A
Sa Cunha et al. [40]	2007	Single-center Retrospective cohort	France	21	Enucleation Distal pancreatectomy Pancreaticoduodenectomy	Laparoscopic vs. OS	9	12	N/A	N/A
Zerbi et al. [41]	2011	Multicenter Prospective cohort	Italy	262	Enucleation Central pancreatectomy Distal pancreatectomy Pancreaticoduodenectomy Total Pancreatectomy	Laparoscopic vs. OS	241	21	N/A	N/A
Zhao et al. [42]	2011	Single-center Retrospective cohort	China	237	Enucleation Central pancreatectomy Distal pancreatectomy Pancreaticoduodenectomy	Laparoscopic vs. OS	191	46	N/A	N/A
Nell et al. [43]	2016	Multicenter Retrospective cohort	Netherlands and France	21	Enucleation Distal pancreatectomy	Robotic vs. Laparoscopic Surgery	N/A	14	7	N/A
Xu et al. [44]	2021	Single-center Retrospective cohort	China	63	Enucleation	MIS vs. OS	11	NR	NR	52
Zheng et al. [45]	2022	Multicenter Retrospective cohort PSM	USA	1.023	Enucleation Central pancreatectomy Distal pancreatectomy Pancreaticoduodenectomy	MIS vs. OS	576	217	230	447

**Table 2:** Summary of findings. MIS: minimally invasive surgery; OS: open surgery; pNETs: pancreatic neuroendocrine tumors; CI: confidence interval; MD: mean difference; RR: risk ratio.

MIS Compared to OS for pNETs				
Study Population: Adults with pNETs Setting: Intraoperative or Postoperative Intervention: MIS Comparison: OS				
Outcomes	Relative Effect (95% CI)	Sample Size (Studies)	Certainty of Evidence (GRADE)	Comments
Severe Complications (Clavien-Dindo Grades 3 to 5 versus Grades 0 to 2)	RR 0.83 (0.61 to 1.12)	1950 participants (7 observational studies)	⨁⨁◯◯ Low ^a,b^	MIS may reduce severe complications compared to OS
Recurrence	RR 0.46 (0.33 to 0.63)	1646 participants (8 observational studies)	⨁⨁⨁⨁ High ^b^	MIS decreases recurrence
R0 Resection	RR 1.06 (0.99 to 1.12)	3360 participants (10 observational studies)	⨁◯◯◯ Very low ^c,d^	MIS may have little to no effect on R0 Resection, but the evidence is very uncertain
Postoperative pancreatic fistula (POPF)	RR 1.05 (0.93 to 1.20)	4340 participants (25 observational studies)	⨁⨁⨁◯ Moderate ^d^	MIS probably has no effect on POPF
Operative time	MD −1.42 (−17.79 to 14.96)	3520 participants (18 observational studies)	⨁◯◯◯ Very low ^c,d^	MIS may have little to no effect on operative time, but the evidence is very uncertain
Length of hospital stay	MD −4.87 (−6.19 to −3.56)	3992 participants (25 observational studies)	⨁⨁◯◯ Low ^c^	MIS may result in a reduction in the length of hospital stay
Intraoperative blood loss	MD −108.47 mL (−177.47 to −39.47)	3505 participants (18 observational studies)	⨁⨁◯◯ Low ^c^	MIS may reduce intraoperative blood loss

^a^ Downgraded by 1 level because the Optimal Information Size was not met. ^b^ Downgraded by 1 level due to small-study effect (publication bias) demonstrated by Funnel plot visual asymmetry. ^c^ Downgraded by 2 levels because of a greater than 75% *I^2^* value. ^d^ Downgraded by 1 level because the CI was wide and did not point to a specific direction of effect.

## Data Availability

The data used to support the findings of this study are available from the corresponding author upon request.

## Supplementary Materials

The following supporting information can be downloaded at: https://www.mdpi.com/article/10.3390/jcm13216303/s1, Figure S1: Forest plots of subgroup analyses: MIS vs. OS for Enucleation, DP, PD procedures; Figure S2: Forest plots of subgroup analyses: Laparoscopic versus OS, Robotic versus OS, and unspecified MIS versus OS; Figure S3: Funnel plots; Figure S4: Contour-enhanced funnel plots; Figure S5: Network maps; Figure S6: Forest plots of network meta-analysis; Figure S7: Rankograms and local inconsistency tests; Table S1: Risk of bias assessment of studies included in this systematic review.
