# Supplementary material for: Robotic Versus Laparoscopic Versus Open Surgery for Non-Metastatic Pancreatic Neuroendocrine Tumors (pNETs): A Systematic Review and Network Meta-Analysis
Source: J Clin Med. 2024 Oct 22;13(21):6303. doi: 10.3390/jcm13216303 (PMC11546742; doi:10.3390/jcm13216303)
Supplement: Supplementary file 1 [file jcm-13-06303-s001.zip › Supplementary_Table_S1.pdf]

Study	Confounding	Participants selection	Classification of interventions	Deviation from intended interventions	Missing data	Measurement of outcomes	Selection of the reported results	Overall risk of bias
Alfieri et al.	Moderate	Low	Low	Low	Low	Low	Moderate	Moderate
Belfiori et al.	Moderate	Low	Low	Low	Low	Low	Moderate	Moderate
Cienfuegos et al.	Moderate	Low	Low	Low	Low	Low	Moderate	Moderate
Fahmy et al.	Moderate	Low	Low	Moderate	Moderate	Low	Serious	Serious
Han et al.	Moderate	Low	Low	Low	Moderate	Low	Moderate	Moderate
Hwang et al.	Low	Low	Low	Low	Low	Low	Low	Low
Kim H et al.	Moderate	Low	Low	Low	Low	Low	Moderate	Moderate
Kim J et al.	Moderate	Low	Low	Low	Moderate	Low	Serious	Serious
Lopez et al.	Moderate	Low	Low	Low	Low	Low	Moderate	Moderate
Mintziras et al.	Moderate	Low	Low	Low	Moderate	Low	Serious	Serious
Naples et al.	Moderate	Low	Moderate	Low	Low	Low	Moderate	Moderate
Partelli et al.	Moderate	Low	Low	Low	Low	Low	Moderate	Moderate
Shiozaki et al.	Moderate	Low	Low	Low	Low	Low	Moderate	Moderate
Sutton et al.	Moderate	Low	Low	Low	Low	Low	Moderate	Moderate
Tan et al.	Low	Low	Low	Low	Low	Low	Low	Low
Tian et al.	Low	Low	Low	Low	Low	Low	Low	Low
Xourafas et al.	Low	Low	Low	Serious	Low	Low	Low	Serious
Zhang J et al.	Moderate	Low	Low	Low	Moderate	Low	Low	Moderate
Zhang X et al.	Low	Low	Low	Low	Low	Low	Low	Low
Espana-Gomez et al.	Moderate	Moderate	Moderate	Serious	Low	Low	Moderate	Serious
Gumbs et al.	Low	Low	Low	Moderate	Moderate	Low	Moderate	Moderate
Hu et al.	Low	Moderate	Low	Moderate	Moderate	Low	Moderate	Moderate
Karaliotas et al.	Moderate	Moderate	Low	Moderate	No Information	Low	No Information	Moderate
Liu et al.	Moderate	Moderate	Low	Low	Serious	Moderate	Moderate	Serious
Lo et al.	Serious	Serious	Low	Low	No Information	Low	Moderate	Serious
Roland et al.	Moderate	Low	Low	Moderate	No Information	Low	Low	Moderate
Sa Cunha et al.	Moderate	Moderate	Low	Serious	Moderate	Low	Low	Serious
Zerbi et al.	Serious	Serious	Low	Low	Low	Low	No Information	Serious
Zhao et al.	Moderate	Moderate	Low	Serious	Moderate	Low	Low	Serious
Nell et al.	Moderate	Moderate	Low	Low	Moderate	Moderate	Low	Moderate
Xu et al.	Moderate	Low	Low	Low	No Information	Low	No Information	Moderate
Zheng et al.	Low	Low	Low	Low	Low	Low	Low	Low
