# Supplementary figures and images for: Robotic Versus Laparoscopic Versus Open Surgery for Non-Metastatic Pancreatic Neuroendocrine Tumors (pNETs): A Systematic Review and Network Meta-Analysis
Source: J Clin Med. 2024 Oct 22;13(21):6303. doi: 10.3390/jcm13216303 (PMC11546742; doi:10.3390/jcm13216303)

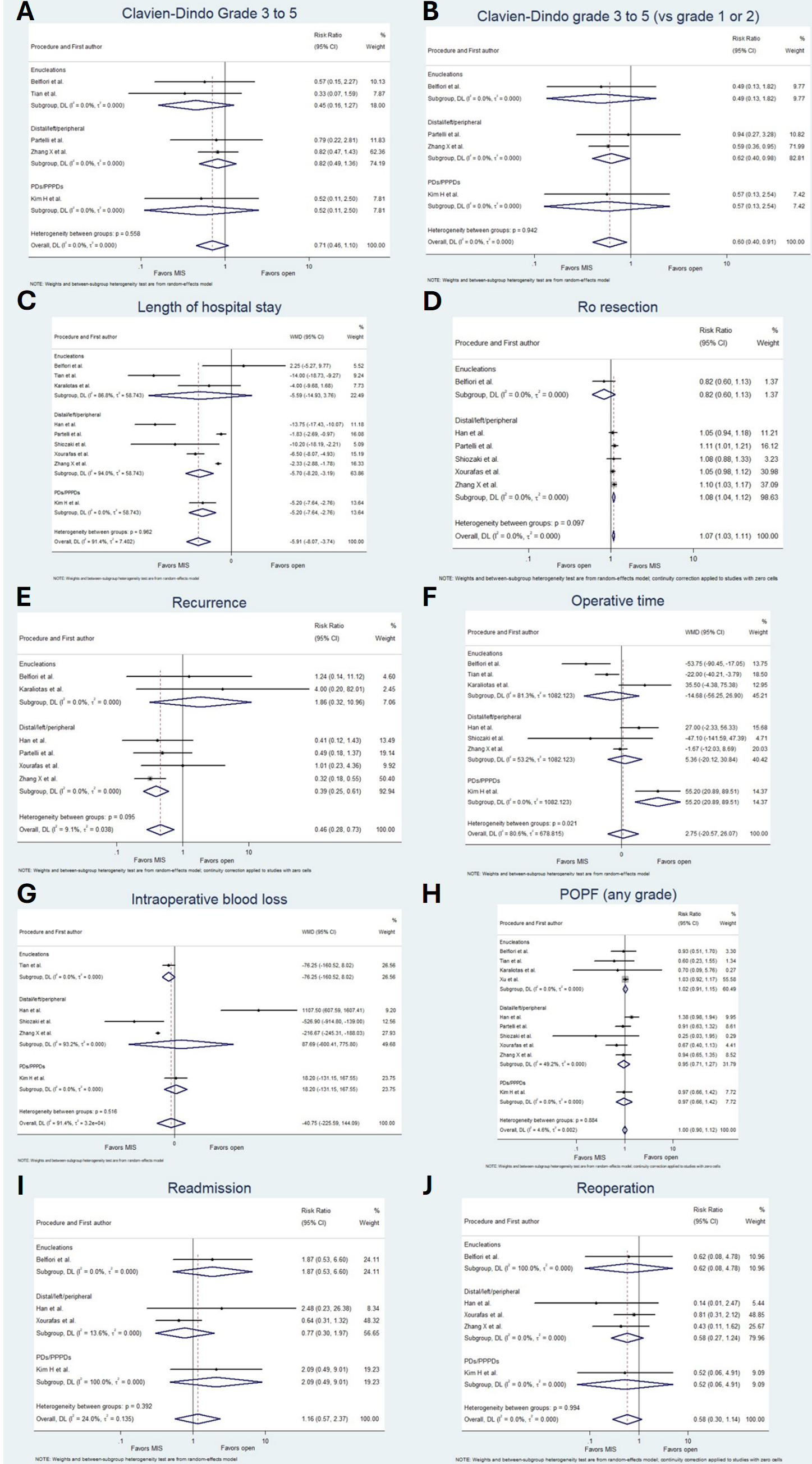

Supplement: Supplementary file 1 [file jcm-13-06303-s001.zip › Supplementary_Figure_S1.tif]

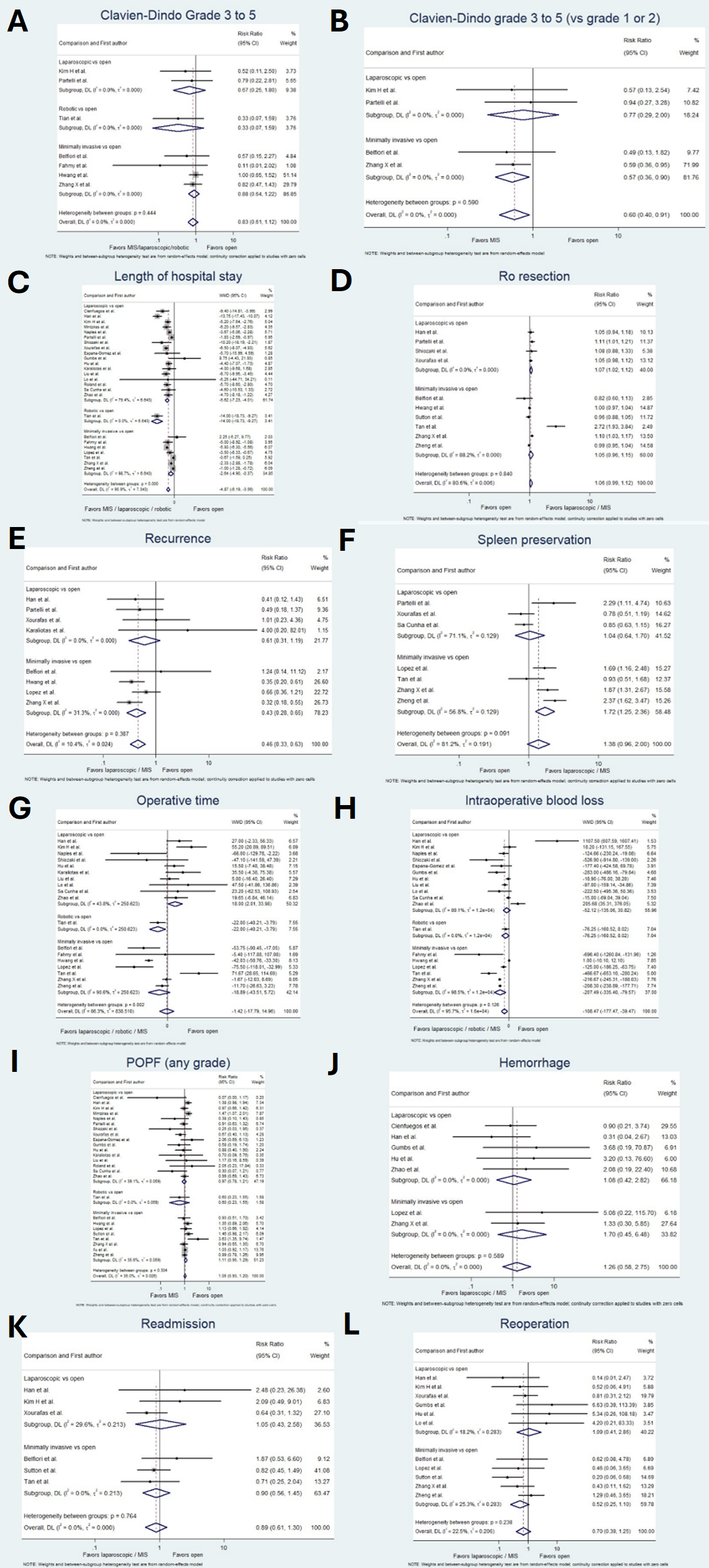

Supplement: Supplementary file 1 [file jcm-13-06303-s001.zip › Supplementary_Figure_S2.tif]

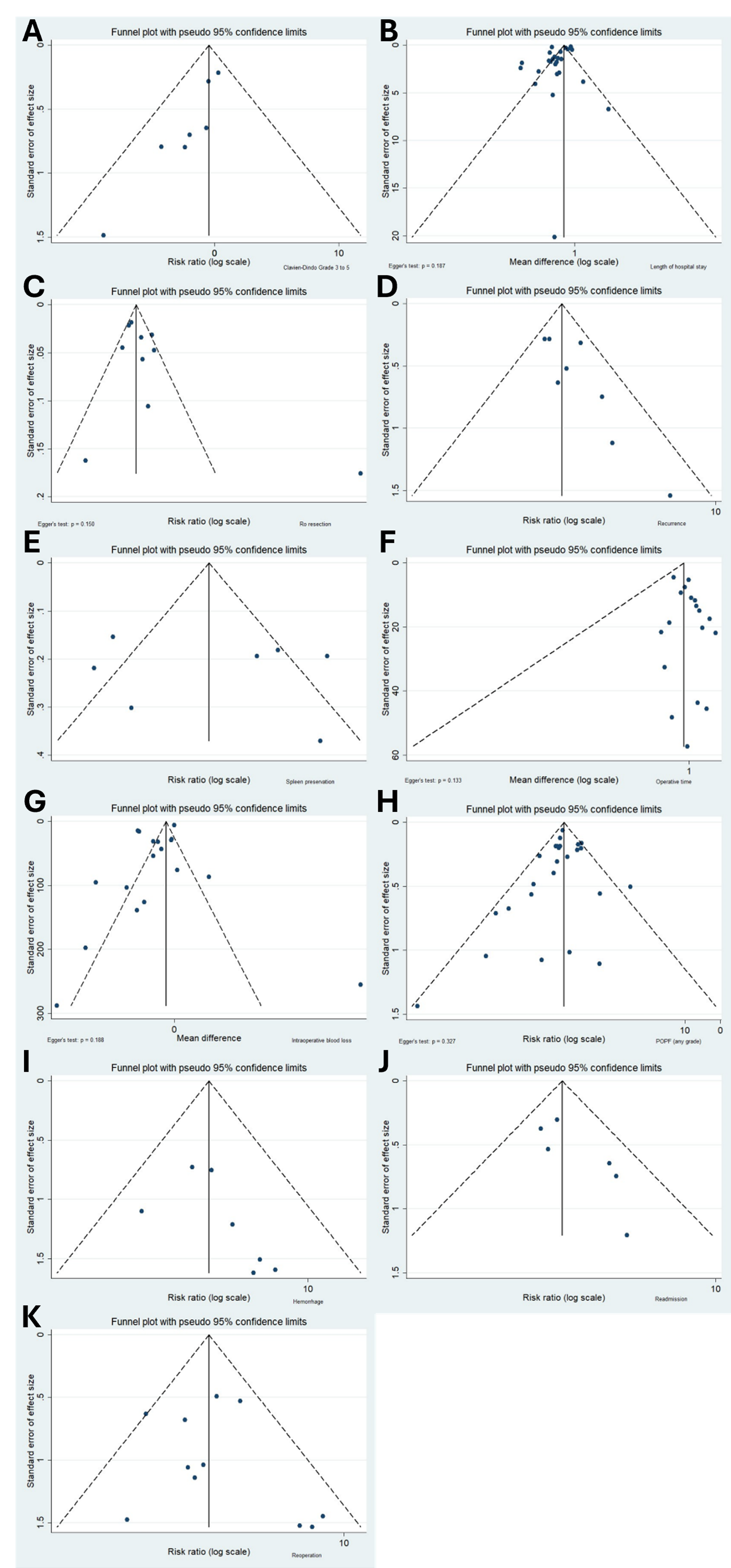

Supplement: Supplementary file 1 [file jcm-13-06303-s001.zip › Supplementary_Figure_S3.tif]

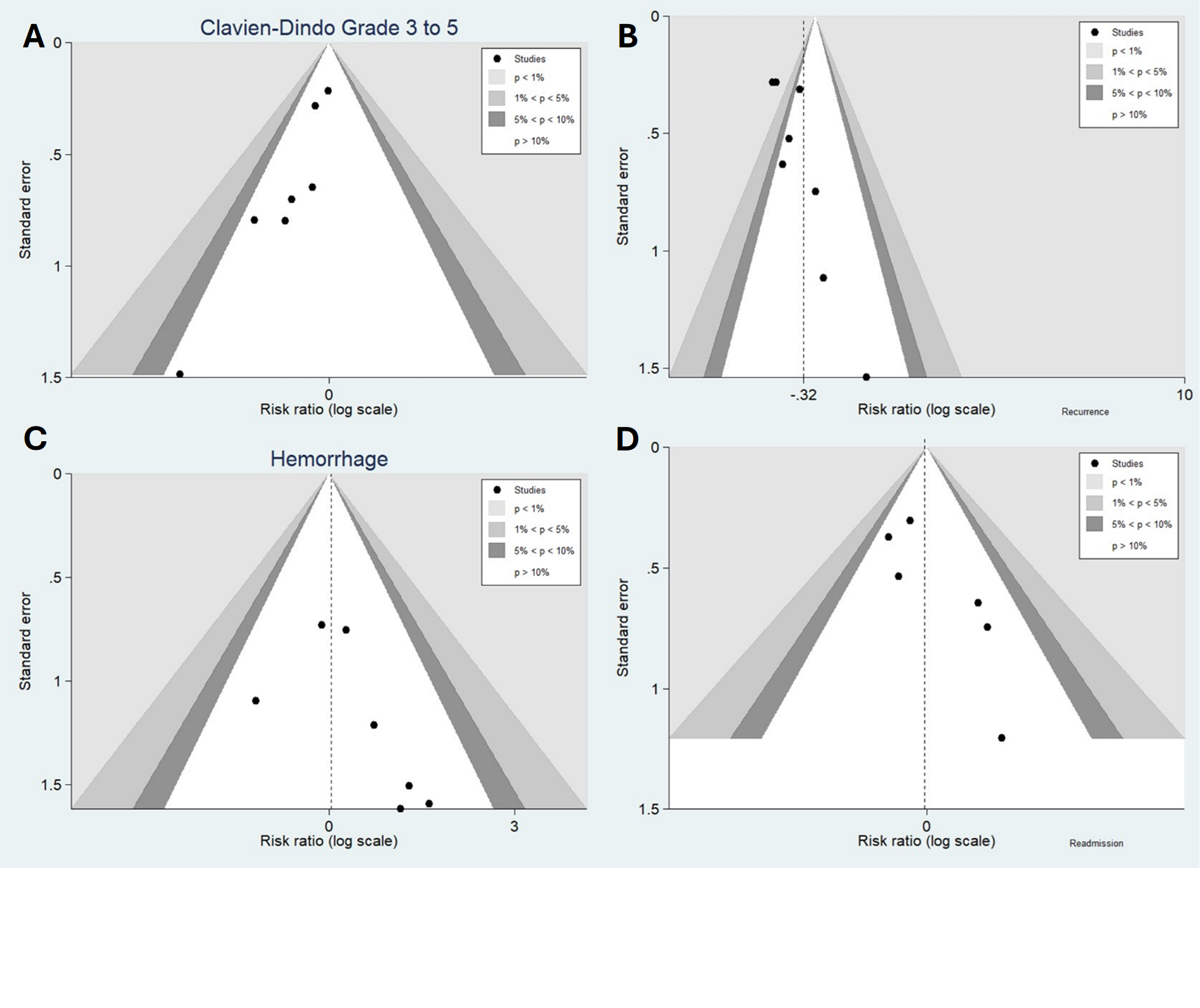

Supplement: Supplementary file 1 [file jcm-13-06303-s001.zip › Supplementary_Figure_S4.tif]

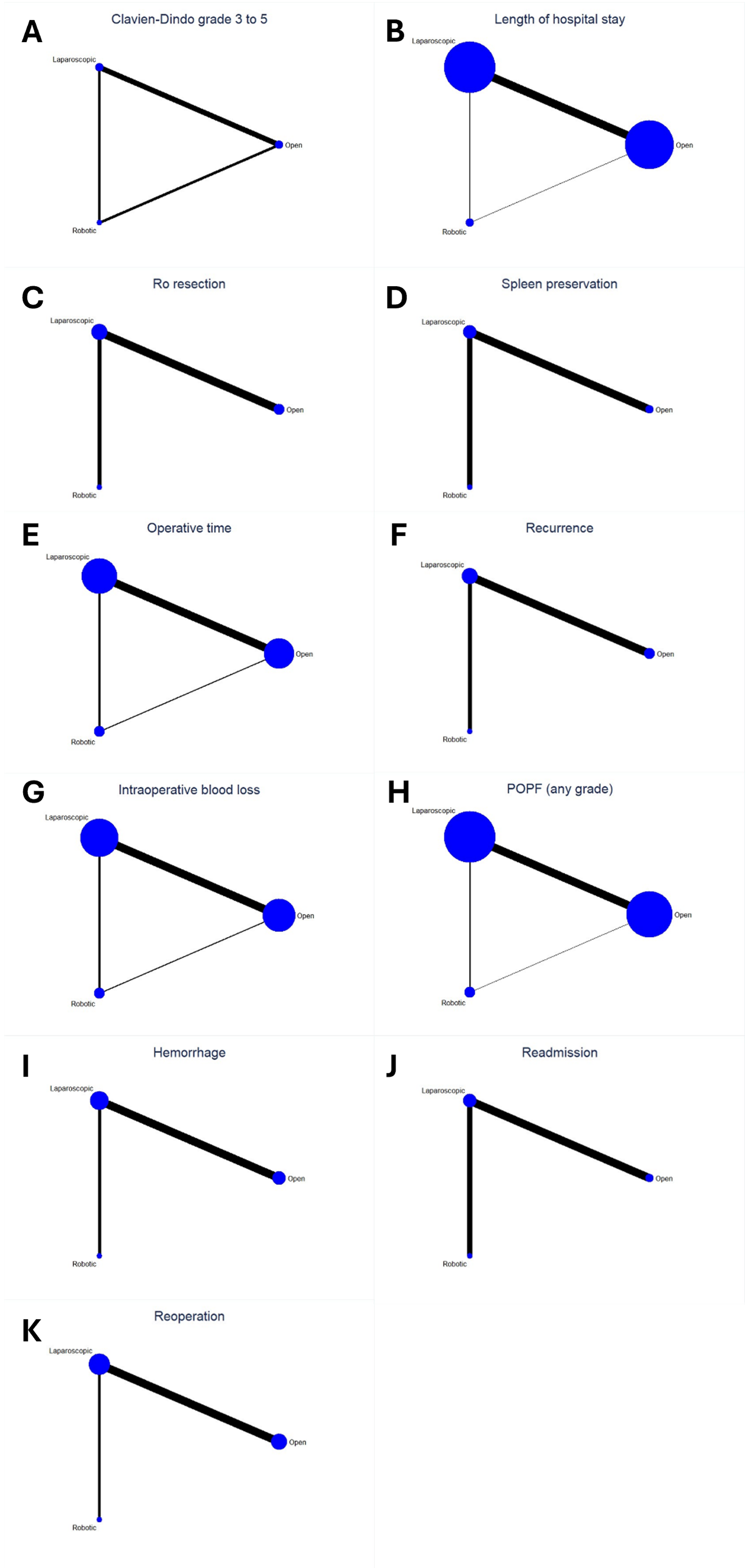

Supplement: Supplementary file 1 [file jcm-13-06303-s001.zip › Supplementary_Figure_S5.tif]

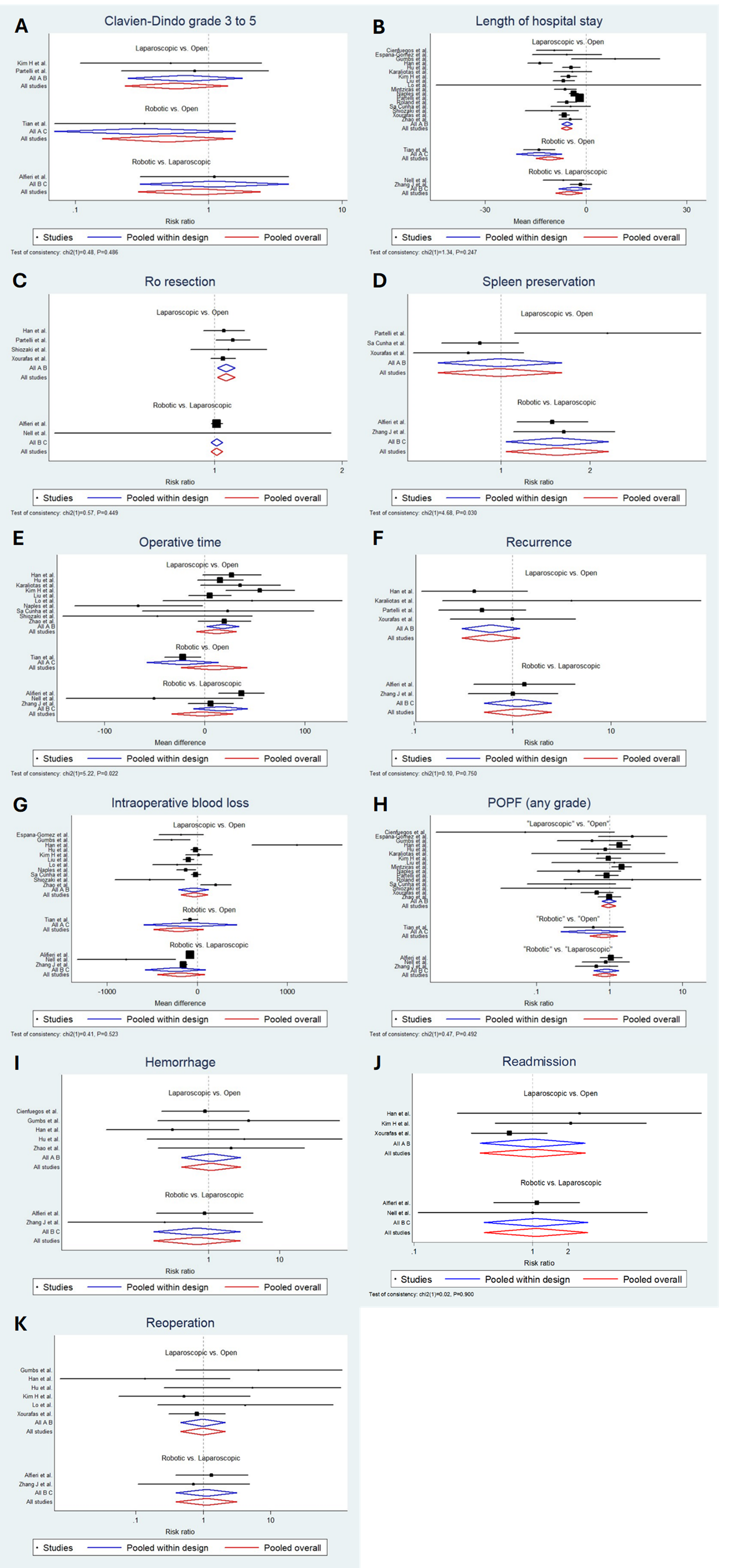

Supplement: Supplementary file 1 [file jcm-13-06303-s001.zip › Supplementary_Figure_S6.tif]

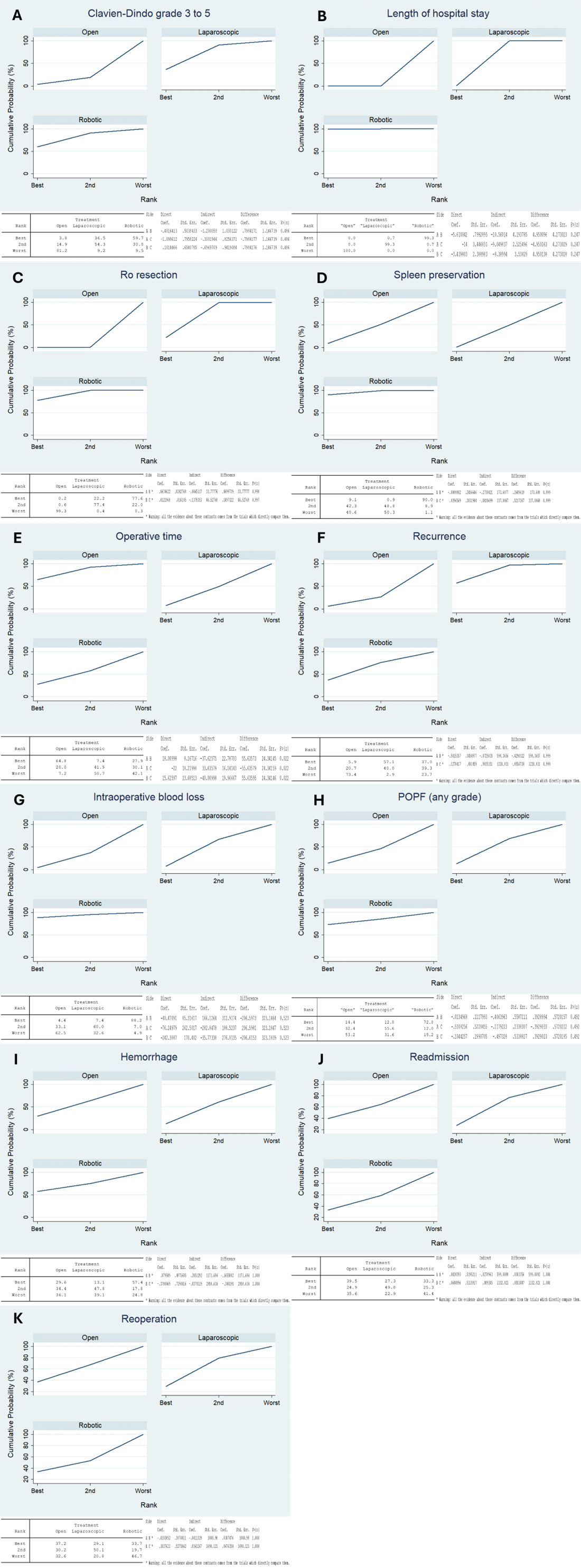

Supplement: Supplementary file 1 [file jcm-13-06303-s001.zip › Supplementary_Figure_S7.tif]
